# Editorial: Combined abiotic interactions in woody plants

**DOI:** 10.3389/fpls.2024.1455459

**Published:** 2024-07-17

**Authors:** Hülya Torun, Claudia Cocozza, Peter Petrík, Srdjan Stojnic

**Affiliations:** ^1^ Department of Biosystem Engineering, Faculty of Agriculture, Düzce University, Düzce, Türkiye; ^2^ Department of Agriculture, Food, Environment and Forestry (DAGRI), University of Florence, Florence, Italy; ^3^ Karlsruhe Institute of Technology (KIT), Institute of Meteorology and Climate Research-Atmospheric Environmental Research, Institut für Meteorologie und Klimaforschung Atmosphärische Umweltforschung (IMK-IFU), Garmisch-Partenkirchen, Germany; ^4^ Chair of Forest Botany, Institute of Forest Botany and Forest Zoology, Technical University of Dresden (TUD), Tharandt, Germany; ^5^ Institute of Lowland Forestry and Environment, University of Novi Sad, Novi Sad, Serbia

**Keywords:** drought, heat stress, heavy metals, light, ROS, temperature, trees

## 1 Introduction

Combined abiotic stress interactions, such as the simultaneous occurrence of drought and high temperatures, significantly impact plants’ physiology, growth, and survival. The occurrence of multiple stressors often exacerbates the effects of each other, leading to compounded challenges for plants (Zandalinas and Mittler, 2022). Additionally, the energy expenditure required to combat multiple stresses can deplete the plant’s reserves, hindering its growth and reproductive capabilities (Maurya et al.). Over time, such compounded stresses can lead to reduced ecosystem productivity, altered species composition, and potentially increased plant mortality rates. Understanding stress interaction effects is essential due to the complexity of climate change, which is increasing both the frequency and severity of droughts and heatwaves across the globe (Tripathy et al., 2023). The impact of these two stressors can have devastating effects on sites already affected by other abiotic stressors such as higher soil salinity, heavy metals or other ecotoxic chemicals. Moreover, the land use legacy can also amplify negative impact on trees, especially in urban ecosystems (Cui et al.). The articles in this Research Topics explored the effects of drought and heat stress, light availability and heavy metal stress on the growth and physiology of trees.

## 2 Drought and heat stress

The co-occurrence of drought and heat stress is one of the most common stress interactions in nature. Drought conditions can limit water availability, reducing the tree’s ability to cool itself through transpiration, while high temperatures can increase water loss and further strain the tree’s water balance. The heat stress can also damage photosynthetic apparatus thus further negatively affecting the carbon balance and growth (Yuan et al.). The heat-drought stress combination can therefore lower water uptake, impair photosynthesis, reduce water-use efficiency, increase reactive oxygen species (ROS) generation, reduce growth and heighten mortality risk. The abiotic stressors can also weaken the tree’s overall health, making it more susceptible to diseases and pests (Teshome et al., 2020). There are multiple options with which we can try to reduce the negative impact of drought and heat stress in forest ecosystems. The application of N can improve the water-use efficiency and growth of drought prone forests in desert regions (Bai et al.). Besides, the exogenous application of plant phytohormones such as auxins, cytokinins, gibberellins, abscisic acid, salicylic acid, jasmonic acid etc. whether prior to or concurrent with the onset of drought and heat stress, has been demonstrated to enhance thermotolerance in plants and to facilitate the maintenance of internal water balance (Paul et al., 2018; Li et al., 2021; Seleiman et al., 2021; Huang et al., 2023). Another important co-factor that can positively improve drought tolerance of forest ecosystems is the selection of drought resistant clones or cultivars (Vuksanović et al.).

## 3 Light, water and temperature

An important factor of the drought-heat interaction in forest ecosystems is the light availability. The incoming light intensity is affected by overall stand structure, tree density and canopy arrangement. The open canopy forests show greater photosynthetic efficiency and growth (Čater et al.), but at the same time provide less shade and therefore less optimal microclimate under drought and heat stress in regard to water balance (Zavadilová et al., 2023). The incoming light quantity also affects the nutrient availability and accumulation in leaves. The trees in forest understory have a higher leaf N/P ratio than ones in forest edges or gaps (Zhang et al.). The higher N/P ratio can positively affect photosynthesis and synthesis of secondary metabolites, but can negatively impact ATP production and overall metabolic activity. The interaction between light availability and abiotic stressors is still under-explored for tree species. New research in this direction is especially needed as thinning, the most common practice in forest management to alter the canopy structure and light regime in forest ecosystems.

## 4 Drought and heavy metal stress

Drought and heavy metal stress interact synergistically in plants, creating a compounded adverse effect on their physiology and growth. Drought conditions limit water availability, causing stomatal closure to reduce transpiration, which in turn restricts CO_2_ intake and diminishes photosynthetic efficiency. Concurrently, heavy metal stress, caused by elements like cadmium, lead, and arsenic, disrupts root function and nutrient uptake, further impairing water absorption and leading to additional dehydration (Sitko et al., 2021). This combination exacerbates nutrient imbalances as heavy metals compete with essential nutrients, such as calcium and zinc, for uptake. Both stresses independently generate ROS, but together they significantly elevate ROS levels, overwhelming the plant’s antioxidant defenses and causing extensive cellular damage (Kostić et al.). Exogenous application of phytohormones or other biotechnological compounds, such as salicylic acid, can alleviate or reduce the negative impact of the combined drought and heavy metal stress (Torun et al.). The understanding of the interaction of heavy metal stress with other abiotic stressors, as well as, the effectivity of biotechnological applications is critical for phytoremediation and land remediation practices.

## 5 Concluding remarks and future perspectives

In conclusion, the interplay between multiple abiotic stressors, such as drought and high temperatures, exerts a profound impact on the physiology, growth, and survival of plants. The simultaneous occurrence of these stressors amplifies their individual effects, presenting compounded challenges that strain the energy reserves of plants and hinder their growth and reproductive success. Understanding these interactions is crucial, especially in the context of climate change, which is intensifying the frequency and severity of droughts and heatwaves globally. The detrimental effects of combined stresses are even more pronounced in environments already compromised by other abiotic factors like soil salinity, heavy metals, or ecotoxic chemicals. Future research should include multi-stress studies with more than two stressors. Moreover, there is a gap in knowledge regarding the impact of seasonal timing (spring vs. summer), severity and stress duration in trees. The research focused on biotechnological applications or breeding with regard to multiple-stressors is also needed to reach effective adaptation strategies for climate change.

## Author contributions

HT: Supervision, Writing – original draft. CC: Supervision, Writing – original draft. PP: Writing – original draft. SS: Supervision, Writing – original draft.

## References

[B1] Huang J. Hartmann H. Ogaya R. Schöning I. Reichelt M. Gershenzon J.. (2023). Hormone and carbohydrate regulation of defense secondary metabolites in a Mediterranean forest during drought. Environ. Exp. Bot. 209, 105298. doi: 10.1016/j.envexpbot.2023.105298

[B2] Li N. Euring D. Cha J. Y. Lin Z. Lu M. Huang L.-J.. (2021). Plant hormone-mediated regulation of heat tolerance in response to Global climate change. Front. Plant Sci. 11, 2020. doi: 10.3389/fpls.2020.627969 PMC7905216 33643337

[B3] Paul S. Wildhagen H. Janz D. Polle A. (2018). Drought effects on the tissue- and cell-specific cytokinin activity in poplar. AoB Plants 10, 1–8. doi: 10.1093/aobpla/plx067 PMC5767954 29354257

[B4] Seleiman M. F. Al-Suhaibani N. Ali N. Akmal M. Alotaibi M. Refay Y.. (2021). Drought Stress Impacts on Plants and Different Approaches to Alleviate Its Adverse Effects. J. Plant. 10 (2), 259. doi: 10.3390/plants10020259 PMC7911879 33525688

[B5] Sitko K. Opała-Owczarek M. Jemioła G. Gieroń Ż. Szopiński M. Owczarek P.. (2021). Effect of drought and heavy metal contamination on growth and photosynthesis of silver birch trees growing on post-industrial heaps. Cells 11, 53. doi: 10.3390/cells11010053 35011615 PMC8750922

[B6] Teshome D. T. Zharare G. E. Naidoo S. (2020). The threat of the combined effect of biotic and abiotic stress factors in forestry under a changing climate. Front. Plant Sci. 11. doi: 10.3389/fpls.2020.601009 PMC7733969 33329666

[B7] Tripathy K. P. Mukherjee S. Mishra A. K. Mann M. E. Williams A. P. (2023). Climate change will accelerate the high-end risk of compound drought and heatwave events. Proc. Natl. Acad. Sci. U.S.A. 120, e2219825120. doi: 10.1073/pnas.2219825120 37399379 PMC10334742

[B8] Zandalinas S. I. Mittler R. (2022). Plant responses to multifactorial stress combination. New Phytol. 234, 1161–1167. doi: 10.1111/nph.18087 35278228

[B9] Zavadilová I. Szatniewska J. Stojanović M. Fleischer P. Vágner L. Pavelka M.. (2023). The effect of thinning intensity on sap flow and growth of Norway spruce. J. For. Sci. 69, 205–216. doi: 10.17221/17/2023-JFS

